# A piperazidine derivative of 23-hydroxy betulinic acid induces a mitochondria-derived ROS burst to trigger apoptotic cell death in hepatocellular carcinoma cells

**DOI:** 10.1186/s13046-016-0457-1

**Published:** 2016-12-08

**Authors:** Nan Yao, Ying-jie Li, Yu-he Lei, Nan Hu, Wei-Min Chen, Zhe Yao, Miao Yu, Jun-shan Liu, Wen-cai Ye, Dong-mei Zhang

**Affiliations:** 1 College of Pharmacy, Jinan University, No.601 West Huangpu Avenue, Guangzhou, 510632 China; 2 School of Traditional Chinese Medicine, Southern Medical University, Guangzhou, 510515 China

**Keywords:** Hepatocellular carcinoma, Betulinic acid, Apoptosis, Reactive oxygen species, Mitochondria

## Abstract

**Background:**

Elevated production of reactive oxygen species (ROS) and an altered redox state have frequently been observed in hepatocellular carcinoma (HCC); therefore, selective killing of HCC cells by chemotherapeutic agents that stimulate ROS generation or impair antioxidant systems may be a feasible approach in HCC chemotherapy. Recently, betulinic acid and its derivatives have attracted attention because they showed anti-cancer effects via a ROS- and mitochondria-related mechanism. However, the source of ROS overproduction and the role of mitochondria were poorly identified, and the weak in vivo antitumour activity of these compounds limits their development as drugs.

**Methods:**

Cytotoxicity was detected using MTT assays. In vivo anti-HCC effects were assessed using nude mice bearing HepG2 tumour xenografts. Cell cycle analysis, apoptosis rate and mitochondrial membrane potential were measured by flow cytometry. ROS production was detected using a microplate reader or a fluorescence microscope. Changes in gene and protein levels were measured by RT-PCR and western blotting, respectively. Other assays were performed using related detection kits.

**Results:**

B5G9, a piperazidine derivative of 23-hydroxy betulinic acid (23-HBA), showed excellent in vivo anti-HCC effects, with a tumour growth inhibitory rate of greater than 80%, and no significant side effects. B5G9 stimulated the production of ROS, which were derived from the mitochondria, but it had no effect on various other antioxidant systems. Moreover, B5G9 induced mitochondrial dysfunction, which was characterized by morphological changes, membrane potential collapse, membrane permeabilization, and decreases in the O_2_ consumption rate and ATP production. Furthermore, mtDNA-depleted ρ0 HepG2 cells were less sensitive to B5G9 treatment than wt HepG2 cells, indicating the importance of mitochondria in B5G9-induced cell death.

**Conclusion:**

We discovered a piperazidine derivative of 23-HBA, B5G9, with excellent anti-HCC effects both in vivo and in vitro and no obvious toxic effects. The underlying mechanism was associated with mitochondria-derived ROS overproduction, and mitochondria played essential roles in B5G9-induced cell death. This study identified a potential agent for anti-HCC therapy and elucidated the mitochondria-related mechanism of BA and its derivatives.

**Electronic supplementary material:**

The online version of this article (doi:10.1186/s13046-016-0457-1) contains supplementary material, which is available to authorized users.

## Background

Hepatocellular carcinoma (HCC), the most common type of liver cancer, is the sixth most common malignancy in the world [1]. The majority of HCC patients are diagnosed at an advanced or even late stage due to the unapparent initial symptoms and rapid progression. Chemotherapy is the primary therapeutic approach against HCC [2]. Chemotherapeutics such as sorafenib, a tyrosine kinase inhibitor, and several other cytotoxic drugs, are front-line anti-HCC drugs. However, these agents are more or less toxic and have variable effects in different patients because of the complicated molecular mechanisms of HCC, leading to decreased mortality of patients [3]. Therefore, novel anti-HCC drugs are urgently needed to improve the survival and prognosis of HCC patients. Bioactive natural products or their semi-synthetic derivatives provide an abundant source for the development of new anti-HCC drugs due to their low toxicity [4].

Reactive oxygen species (ROS), a by-product of the normal metabolism of oxygen, play an important role in cell proliferation and differentiation [5]. However, during environmental stress, cellular ROS levels can increase dramatically, which results in substantial damage to cellular physiology and subsequent cell death [6]. Accumulating evidence has suggested that cancer cells exhibit increased levels of ROS compared with those of the parental cells due to oncogenic stimulation, increased metabolic activity and mitochondrial dysfunction [7, 8]. Cancer cells are more sensitive to the excessive oxidative stress induced by ROS-generating agents or antioxidant enzyme inhibitors. Therefore, selectively killing cancer cells using ROS-elevating agents is an effective approach in cancer chemotherapy.

In normal physiological conditions, cellular ROS is maintained at a stable level due to the dynamic balance between ROS production and elimination. ROS overproduction or antioxidant system impairment may result in oxidative stress [9]. Mitochondria are the primary source of endogenous ROS [10]. Under normal conditions, 1–2% of electrons escape from the mitochondrial electron transport chain (ETC) and then interact with singlet oxygen to form superoxide anions, the initial form of ROS. However, when the ETC is disturbed by environmental stimulation or mitochondria are impaired, electrons increasingly combine with singlet oxygen, leading to excess ROS [11, 12]. In addition to mitochondria, ROS are formed in the cytoplasm and plasma membrane. NADPH oxidase, a membrane-bound enzyme, catalyses superoxide anion production via a 1-electron reduction of molecular oxygen [13]. Other cytoplasmic enzymes, including xanthine oxidase, cyclooxygenase, lipoxygenase, and cytochrome p450, also participate in the generation of ROS via reactions between electrons and molecular oxygen [14]. Impairment of antioxidant systems, including antioxidant enzymes and non-enzymatic ROS scavengers, may disturb the balance of ROS homeostasis and trigger increased ROS production. Several antioxidant inhibitors, such as GSH inhibitors (e.g., PEITC), superoxide dismutase (SOD) inhibitors (e.g., ATN-224) and Trx inhibitors (e.g., PX-12), have shown potential antitumour activity associated with oxidative stress [15–17].

Betulinic acid (BA), a naturally occurring pentacyclic triterpenoid isolated from white birch trees, has attracted widespread attentions due to its multiple biological activities, such as anti-HIV, anti-cancer, anti-malarial and anti-inflammatory effects [18, 19]. In vitro studies have shown that BA and its analogue 23-hydroxy betulinic acid (23-HBA) exhibited anti-cancer activity against a wide variety of cancer cells, including melanoma, prostate carcinoma, lung carcinoma, and leukaemia [20–25]. To enhance the benefits and minimize the side effects of this treatment, several novel derivatives have been synthesized. Multiple studies have demonstrated that the antitumour mechanisms of BA, 23-HBA and their derivatives may be related to the mitochondria and ROS [26–30]. However, the underlying mechanisms are unclear, and the source of ROS is still unknown. Thus, exploring the mechanisms of ROS production and the relationship between mitochondrial dysfunction and ROS production would provide an in-depth understanding of the antitumour mechanisms of BA, 23-HBA and their derivatives. Recently, several in vivo antitumour experiments assessing 23-HBA derivatives against H22 liver cancer and B16 melanoma have been reported [25, 31–33], but these derivatives showed weak antitumour activity. Therefore, the development of novel 23-HBA derivatives with excellent in vivo antitumour activity is urgently needed.

In this study, we found that a piperazidine derivative of 23-HBA, B5G9, exhibited excellent in vivo anti-HCC efficiency, with a tumour growth inhibitory rate of more than 80%, and no significant side effects in B5G9-treated nude mice bearing HepG2 xenografts. Moreover, the underlying mechanism of B5G9 was associated with apoptosis induction, and we further explored the relationship among the mitochondria, ROS and apoptosis.

## Methods

### Reagents and antibodies

B5G9 with a purity of 98% was synthesized as described previously [24]. Hoechst 33342, H_2_DCFDA, MitoTracker® Red CMXRos, MitoSOX Red, a BCA protein assay kit and a Dead Cell Apoptosis Kit were purchased from Thermo Fisher Scientific (Waltham, MA, USA). Necrostatin-1 was obtained from Selleck Chemicals (Houston, TX, USA). MnTBAP was purchased from Santa Cruz Biotechnology (Dallas, TX, USA). An antibody against cytochrome *c* was obtained from Epitomics (Burlingame, CA, USA). Antibodies against caspase-3, caspase-9, cleaved-caspase-3, cleaved-caspase-9, PARP and β-actin were obtained from Cell Signaling (Beverly, MA, USA). Other reagents were purchased from Sigma Aldrich (St. Louis, MO, USA).

### Cell culture

The HCC cell lines HepG2 and Hep3B were obtained from the American Type Culture Collection (ATCC, Rockville, MD, USA). Bel-7402 cells were purchased from the Type Culture Collection of the Chinese Academy of Sciences (Xuhui, Shanghai, China). HepG2/ADM cells were kindly provided by Prof. Kwok-Pui Fung (The Chinese University of Hong Kong, Hong Kong, China). The HepG2, Hep3B, HepG2/ADM and Bel-7402 cells were maintained in RPMI 1640 medium (Thermo Fisher Scientific, Waltham, MA, USA) containing 10% (v/v) foetal bovine serum (Thermo Fisher Scientific, Waltham, MA, USA) and 1% (v/v) penicillin-streptomycin (PS; 10,000 U/ml, Thermo Fisher Scientific, Waltham, MA, USA) at 37 °C in a 5% CO_2_ atmosphere.

### Cell viability assay

HepG2, HepG2/ADM, Hep3B and Bel-7402 cells (1 × 10^4^/well) were seeded in 96-well plates and cultured overnight. Then, the cells were treated with different concentrations of B5G9 for an additional 12 h, 24 h or 36 h. Subsequently, the cells were incubated with 30 μl of MTT (5 mg/ml) for 4 h. The formazan crystals that formed were solubilized in 100 μl DMSO, and the absorbance was measured at 595 nm using a microplate reader (Beckman Coulter, Brea, CA, USA). Cell viability was calculated as a percentage of the vehicle control (treatment with medium containing 0.2% DMSO).

### Colony formation assay

HepG2 cells were seeded in 6-well microplates at a density of 300 cells per well and cultured overnight. The cells were then treated with various concentrations of B5G9 for 24 h and maintained in fresh medium in an incubator of 5% CO_2_ at 37 °C for 10 days. Next, the cells were fixed in methanol at -20 °C for 10 min and stained with 1% crystal violet for 20 min. Finally, the visible colonies were manually counted.

### Tumour xenografts in nude mice

Six-week-old nude mice were obtained from Vital River Laboratory Animal Technology Co, Ltd. (Beijing, China). All animals were maintained in specific pathogen free (SPF) room. The nude mice subcutaneously inoculated with 1.5 × 10^7^ HepG2 cells. Two weeks later, the mice with the volume of the tumour achieved about 200 mm^3^ were randomly divided into four groups (seven per group): vehicle, B5G9 (20 mg/kg and 40 mg/kg) and 23-HBA (40 mg/kg). The drugs were administered via intragastric injection every day. The vehicle group was administered 0.9% NaCl. Body weight and tumour volume were measured every other day, and tumour volume was calculated as (a × b^2^)/2, where a and b are the longest and the shortest diameters of the tumours, respectively [34]. After 23 days of treatment, tumour volume of mice in the vehicle group reached about 2000 mm^3^, the mice were sacrificed and the tumours, organs and blood were collected for subsequent measurement.

### Histological examination

For histological examination, haematoxylin-eosin (H&E) staining of the viscera was performed as described previously [35]. The images were photographed using an Olympus IX70 inverted microscope (Olympus, Tokyo, Japan).

### Serum biochemistry and routine blood detection

After B5G9 treatment, the blood of the mice was collected and serum biochemistry and routine blood analyses were carried out as described previously [36, 37].

### Hoechst 33342 staining assay

HepG2 cells treated with B5G9 for 12 h were stained with Hoechst (5 μg/ml) 33342 in the dark at 37 °C for 20 min. After being washed with phosphate-buffered saline (PBS), the cells were observed using a fluorescence microscope (Carl Zeiss, Göttingen, Germany).

### Ultrastructure observation by transmission electronic microscopy

HepG2 cells treated with B5G9 for 12 h were fixed in 3% glutaraldehyde overnight and in 1% osmium tetroxide for an additional 1 h. The cells were then dehydrated in a gradient of 50–100% ethanol and polymerized with epoxy resin. The ultra-thin sections were stained with aqueous uranyl acetate and lead citrate. The ultrastructure of the cells was observed using a JEM-1400 Plus transmission electron microscope (JEOL, Tokyo, Japan).

### Cell cycle analysis

HepG2 cells treated with various concentrations of B5G9 were collected and fixed in ice-cold 70% (v/v) ethanol at 4 °C overnight. Then, the cells were resuspended in PBS containing PI (10 μg/ml) and RNase (0.1 mg/ml) and incubated at 37 °C for 30 min in the dark. DNA content was measured with an Epics XL Flow Cytometer (Beckman Coulter, Brea, CA, USA) (Ex = 488 nm and Em = 620 nm).

### Annexin V-FITC/PI staining assay

HepG2 cells treated with various concentrations of B5G9 were collected and stained with detection buffer containing Annexin V-FITC and PI for 15 min at 37 °C in the dark. The fluorescence was detected with an Epics XL Flow Cytometer (Beckman Coulter, Brea, CA, USA) (Ex = 488 nm and Em = 525 nm for Annexin V-FITC; Ex = 488 nm and Em = 620 nm for PI).

### Measurement of intracellular ROS

HepG2 cells were treated with B5G9 for the indicated times, and the cells were stained with H_2_DCFDA (10 μM) in the dark at 37 °C for 20 min. The fluorescence intensity was observed with a fluorescence microscope or detected by a microplate reader (Beckman Coulter, Brea, CA, USA).

### Lipid peroxidation detection

A Lipid Peroxidation MDA Assay Kit (Beyotime, Shanghai, China) was used for the detection of cellular lipid peroxidation. Methane dicarboxylic aldehyde (MDA) was used as a characteristic product of lipid peroxidation. In brief, cell lysates from HepG2 cells treated with B5G9 or the MDA standards were mixed with thiobarbituric acid (TBA) working solution according to the manufacturer’s manual. The absorbance at 535 nm of the MDA-TBA complex was detected with a microplate reader (Beckman Coulter, Brea, CA, USA). The concentration of cellular MDA was calculated according to the standard curve.

### Detection of mitochondrial membrane potential (*ΔΨm*) and mitochondrial permeability transition (MPT)

For *ΔΨm* detection, HepG2 cells treated with B5G9 were stained with JC-1 at 37 °C for 20 min, and the JC-1 fluorescence was detected with an Epics XL Flow Cytometer (Beckman Coulter, Brea, CA, USA). The JC-1 fluorescence conversion from red to green demonstrates the ΔΨm collapse. For the measurement of MPT, a mitochondrial permeability transition pore detection kit (Genmed, Shanghai, China) was used according to the manufacturer’s manual.

### Mitochondrial morphology observation

HepG2 cells were stimulated with B5G9 for the indicated times, and cells were stained with MitoTracker® Red CMXRos at 37 °C for 30 min. Mitochondrial morphology was observed with a fluorescence microscope (Carl Zeiss, Göttingen, Germany).

### Measurement of O_2_ consumption rate

The O_2_ consumption rate (OCR) was measured using an Oxygen Consumption Rate Assay Kit (Cayman Chemical, Ann Arbor, MI, USA). HepG2 cells (20,000/well) were seeded in a 96-well plate and cultured overnight. The cells were treated with B5G9 for the indicated times or antimycin A for 3 h. Then, MitoXpress-Xtra was added and HS Mineral Oil was overlaid in each well. Finally, the MitoXpress-Xtra fluorescence was detected with an Infinite F500 microplate reader (TECAN, Männedorf, Switzerland) using time-resolved fluorescence measurement (delay time: 30 μs; integration time: 100 μs).

### Measurement of cellular ATP

ATP content was determined using a luciferase-dependent method. In brief, HepG2 cells seeded in 96-well plates (20,000/well) were treated with B5G9 in complete medium or no-glucose medium and washed with PBS twice. Cellular ATP content was detected with a Cell Titer-Glo 2.0 Assay kit following the manufacturer’s protocol (Promega, Madison, WI, USA).

### Generation of mtDNA-depleted ρ0 HepG2 cells

ρ0 HepG2 cells were derived from wt HepG2 cells by maintaining the cells in EB (100 ng/ml), pyruvate (100 μg/ml) and uridine (50 μg/ml) for more than 30 generations [38, 39]. Mitochondrial complex I, II, III, IV and ATP synthetase activity assays (Cayman chemical, Ann Arbor, MI, USA) and ATP measurements were conducted to confirm the authenticity of the ρ0 HepG2 cells.

### Western blotting

For total protein extraction, HepG2 cells treated with various concentrations of B5G9 were harvested, washed twice with ice-cold PBS and lysed in RIPA (Thermo Fisher Scientific, Waltham, MA, USA) buffer containing PMSF, phosphatase inhibitors and protease inhibitors for 30 min on ice. After centrifugation at 20,000 g at 4 °C for 10 min, the supernatants were quantified using a BCA protein assay kit. The cytoplasmic and mitochondrial proteins were extracted using a digitonin-based method as described previously [40]. For western blotting, proteins (50 μg per sample) were separated by SDS-PAGE and transferred to a polyvinylidene difluoride (PVDF) membrane (Millipore Corporation, Billerica, MA, USA). Then, the membranes were probed with specific antibodies using standard procedures.

### RT-PCR and RT^2^ Profiler PCR Arrays analysis

Total RNA from HepG2 cells was extracted using an RNeasy Mini Kit (QIAGEN, Hilden, Germany). Reverse transcription was performed using a Transcriptor First Strand cDNA Synthesis Kit (Roche, Mannheim, Germany) with total RNA (2 μg) as described by the manufacturer. Then, RT-PCR was carried out by adding SYBR Green I Master (10 μl) (Roche, Mannheim, Germany), forward primer (0.5 μM), reverse primer (0.5 μM), cDNA (2 μl) and distilled water (6 μl) per sample, and the PCR products were quantified with a LightCycler 480 PCR system (Roche, Mannheim, Germany). To analyse the related genes in B5G9-induced oxidative stress, RT^2^ Profiler PCR Arrays (Human Oxidative Stress, PAHS-065Z, QIAGEN, Hilden, Germany) were used. Primer sequences are shown in Additional file 1: Table S1.

### Statistical analysis

Each experiment was performed for at least three times and data are shown as the mean ± standard deviation. Statistical analysis was performed using Graphpad Prism 4.0 by one-way ANOVA and Tukey post hoc test. A difference was considered significant when *P* < 0.05.

## Results

### In vitro and in vivo anti-HCC activities of B5G9

**Fig. 1:**
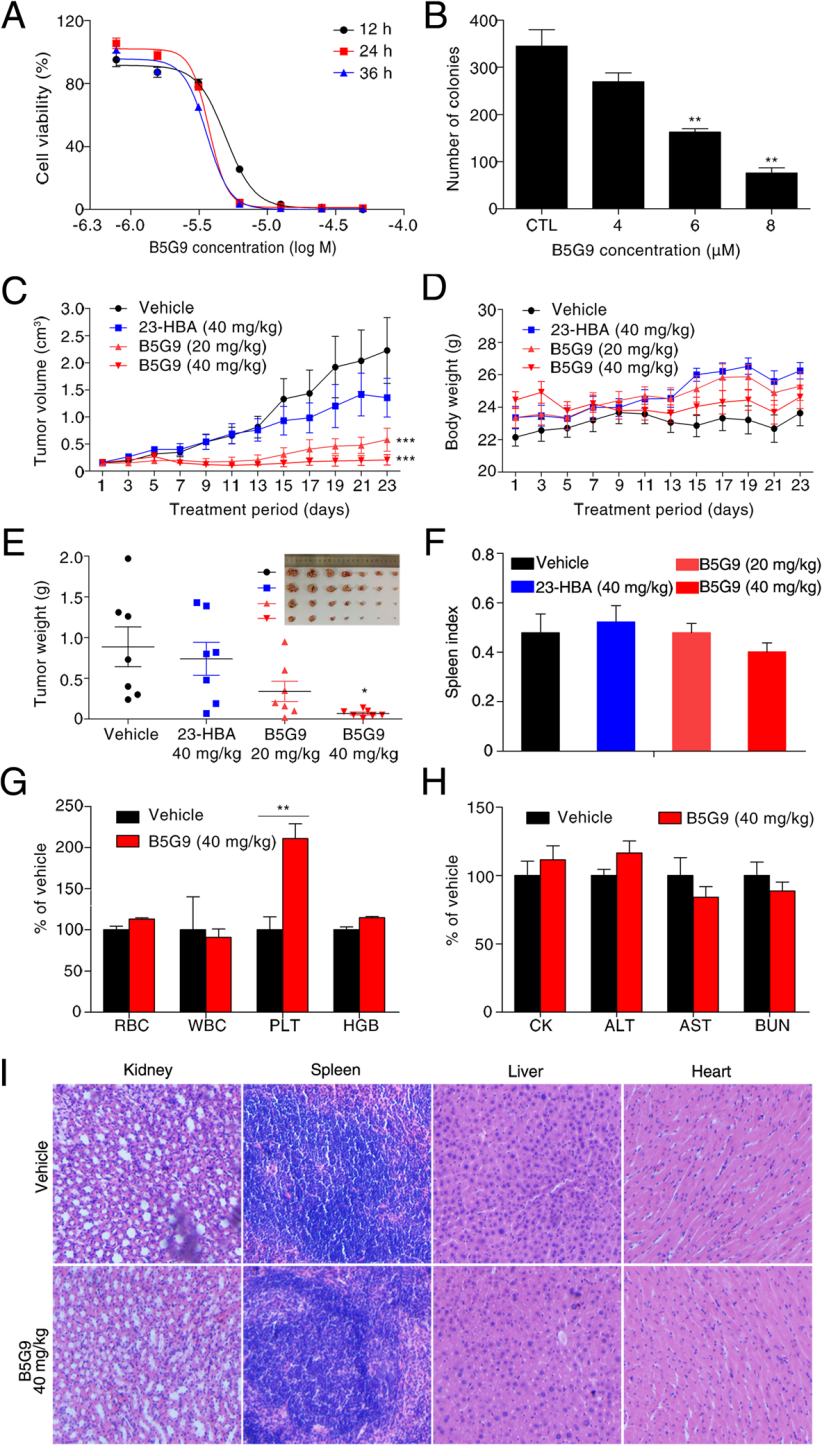
B5G9 suppresses HepG2 cells in vitro and in vivo. **a** The cytotoxicity of B5G9 on HepG2 cells. HepG2 cells were treated with different concentrations of B5G9 for 12, 24 and 36 h. Cell viability was measured by MTT assay. **b** The inhibitory effect of B5G9 on the colony formation of HepG2 cells. HepG2 cells were treated with different concentrations of B5G9 for 24 h. Clonogenic survival of HepG2 cells after B5G9 treatment was measured by the number of clones capable of anchorage-dependent growth. ^**^ *P* ≤ 0.01 *vs* control*.* **c** The growth curves of HepG2 xenografts. Nude mice bearing HepG2 xenografts were treated with B5G9 (20 or 40 mg/kg/day), 23-HBA (20 mg/kg/day) for 23 days. Tumor size was measured every other day. ^***^ *P* ≤ 0.001 *vs* vehicle*.* **d** The body weight curves of the mice measured every 2 days. **e** Tumor weights of HepG2 xenografts dissected after 23 days treatment and the photograph of the tumours isolated from nude mice. ^*^ *P* ≤ 0.05 *vs* vehicle. **f** B5G9 had no effect on the spleen index. **g-h** B5G9 had no significant effect on routine blood indices and serum biochemistrical indices. After 23 days treatment, the mice were killed and blood was collected. Routine blood indices (RBC, WBC, PLT, HGB) and serum biochemistrical indices (CK, AST, ALT and BUN) were measured, ^**^ *P* ≤ 0.01. **i** B5G9 had no influence on the function of kidney, spleen, liver and heart. After 23 days treatment, viscera were taken out for H&E staining

The in vitro anti-HCC activity of B5G9 was evaluated using MTT assays against four HCC cell lines, HepG2, HepG2/ADM, Hep3B and Bel-7402. As shown in Table 1, B5G9 exhibited excellent cytotoxicity against these four HCC cell lines, whereas 23-HBA had a weak effect. Among the cell lines, HepG2 cells were most sensitive to B5G9. B5G9 markedly inhibited the survival of HepG2 cells in a dose-dependent manner (Fig. 1a). The colony formation assay also confirmed that B5G9 inhibited the proliferation of HepG2 cells (Fig. 1b). To determine whether B5G9 exerts anti-HCC activity in vivo, we treated nude mice bearing HepG2 tumour xenografts with vehicle, 23-HBA (40 mg/kg), B5G9 (20 mg/kg) or B5G9 (40 mg/kg) via intragastric administration once every day for 23 days. Tumour volumes were measured every other day. As shown in Fig. 1c, B5G9 (20 mg/kg or 40 mg/kg) had a significant inhibitory effect on the growth of tumours, whereas its parent compound, 23-HBA, had a minimal inhibitory effect. At the end of the treatment, the tumours were isolated and photographed (the photo localized in top right corner in Fig. 1e). The tumour weights of the 20 and 40 mg/kg B5G9-treated mice were 0.34 ± 0.12 g and 0.07 ± 0.02 g, respectively, which were significantly lower than that of the vehicle group (0.89 ± 0.24 g) (Fig. 1e). During the treatment period, B5G9 had no significant effect on the body weight of the mice (Fig. 1d) or the spleen index (Fig. 1f), indicating that B5G9 was well tolerated in mice. To further evaluate the toxicity and side effects of B5G9, we collected mice blood and organs and conducted serum biochemistry and routine blood analyses. As indicated in Fig. 1g, routine blood indices, including measures of white blood cells (WBCs), red blood cells (RBCs), platelets (PLTs) and haemoglobin (HGB), exhibited no significant changes except PLTs. The serum biochemical indices, including creatine kinase (CK), alanine transaminase (ALT), aspartate aminotransferase (AST) and blood urea nitrogen (BUN), also showed no marked changes upon B5G9 treatment (Fig. 1h). The H&E staining of the kidney, spleen, liver and heart also showed that B5G9 had no significant toxic effects on the major organs of mice (Fig. 1i).

**Table 1:** The cytotoxicity of B5G9 in HepG2, HepG2/ADM, Hep3B and Bel-7402 hepatoma carcinoma cell lines

Compounds	IC_50_ (μM) (24 h)			
	HepG2	HepG2/ADM	Hep3B	Bel-7402
B5G9	3.73 ± 0.25	9.94 ± 0.50	10.00 ± 0.55	13.80 ± 0.65
23-HBA	30.35 ± 2.76	40.67 ± 3.73	39.78 ± 3.56	42.78 ± 4.03

HepG2, HepG2/ADM, Hep3B and Bel-7402 cells were treated with different concentrations of B5G9 and 23-HBA for 24 h. Cell viability was measured by MTT assay and IC_50_ values were calculated according to the MTT curves

### HepG2 cells undergo apoptotic cell death upon B5G9 treatment

**Fig. 2:**
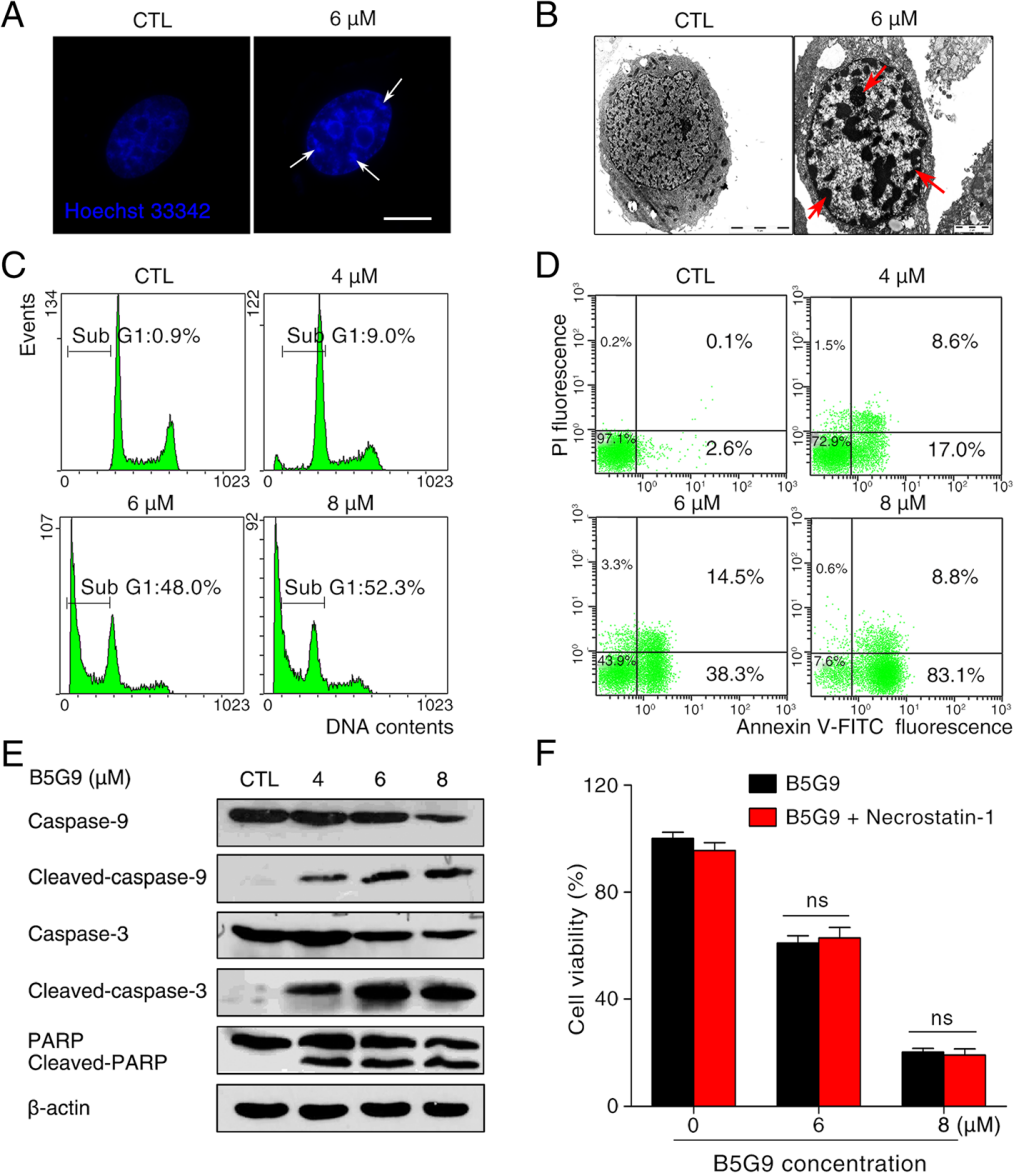
B5G9 induces apoptotic cell death in HepG2 cells. **a** HepG2 cells treated B5G9 (6 μM) were stained with Hoechst 33342 (10 μg/mL) and then observed under a fluorescence microscope. Apoptotic cells with chromatin shrinking were observed (as indicated by the arrow). Original magnifications: × 630; scale bar: 10 μm. **b** The ultrastructure of HepG2 cells after B5G9 (6 μM) treatment for 24 h was observed by transmission electron microscopy. Original magnifications: × 8900; scale bar: CTL, 5 μm; B5G9 (6 μM), 2 μm. **c** B5G9 triggered the accumulation of HepG2 cells in the sub-G_1_ phase of the cell cycle. After B5G9 treatment, HepG2 cells were stained with PI (0.02 mg/mL). Cell cycle distribution was determined by flow cytometry. **d** Apoptotic cells after B5G9 treatment were quantified by the Annexin V/PI assay. HepG2 cells treated with various concentrations of B5G9 were stained using an Annexin V/PI kit and detected by flow cytometry. **e** Effects of B5G9 on the apoptosis-related proteins level. Total cell lysate from HepG2 cells treated with B5G9 at indicated concentrations for 24 h was evaluated by western blotting, and β-actin was used as a loading control. **f** B5G9-induced cell death is necrosis-independent. HepG2 cells were cultured with various concentrations of B5G9 in the presence or absence of necrostatin-1 (50 μM) for 12 h. Cell viability was measured by the MTT assay. The results were presented as the mean ± S.D

Next, we investigated the role of apoptosis in B5G9-induced cell death. Both Hoechst 33342 staining assay and cellular ultrastructure observation demonstrated the apoptotic characteristics in cells treated with B5G9 (as indicated by arrows, Fig. 2a, b). Apoptotic cell death was further confirmed by DNA content analysis as demonstrated by accumulation in the sub G_1_ phase (Fig. 2c). Annexin-V-FITC/PI staining assays were carried out to detect the apoptotic rate, as shown in Fig. 2d, and B5G9 induced apoptosis in a dose-dependent manner. In addition, apoptosis-related proteins were detected by western blot analysis. B5G9 induced the activation of caspase-3 and caspase-9 as well as the cleavage of PARP (Fig. 2e). Moreover, necrostatin-1, a specific inhibitor of necroptosis, had no effect on B5G9-induced cell death (Fig. 2f). The above data indicated that apoptosis, but not necroptosis, is the major process involved in B5G9-induced cell death.

### B5G9 induced apoptosis in a ROS-dependent manner

**Fig. 3:**
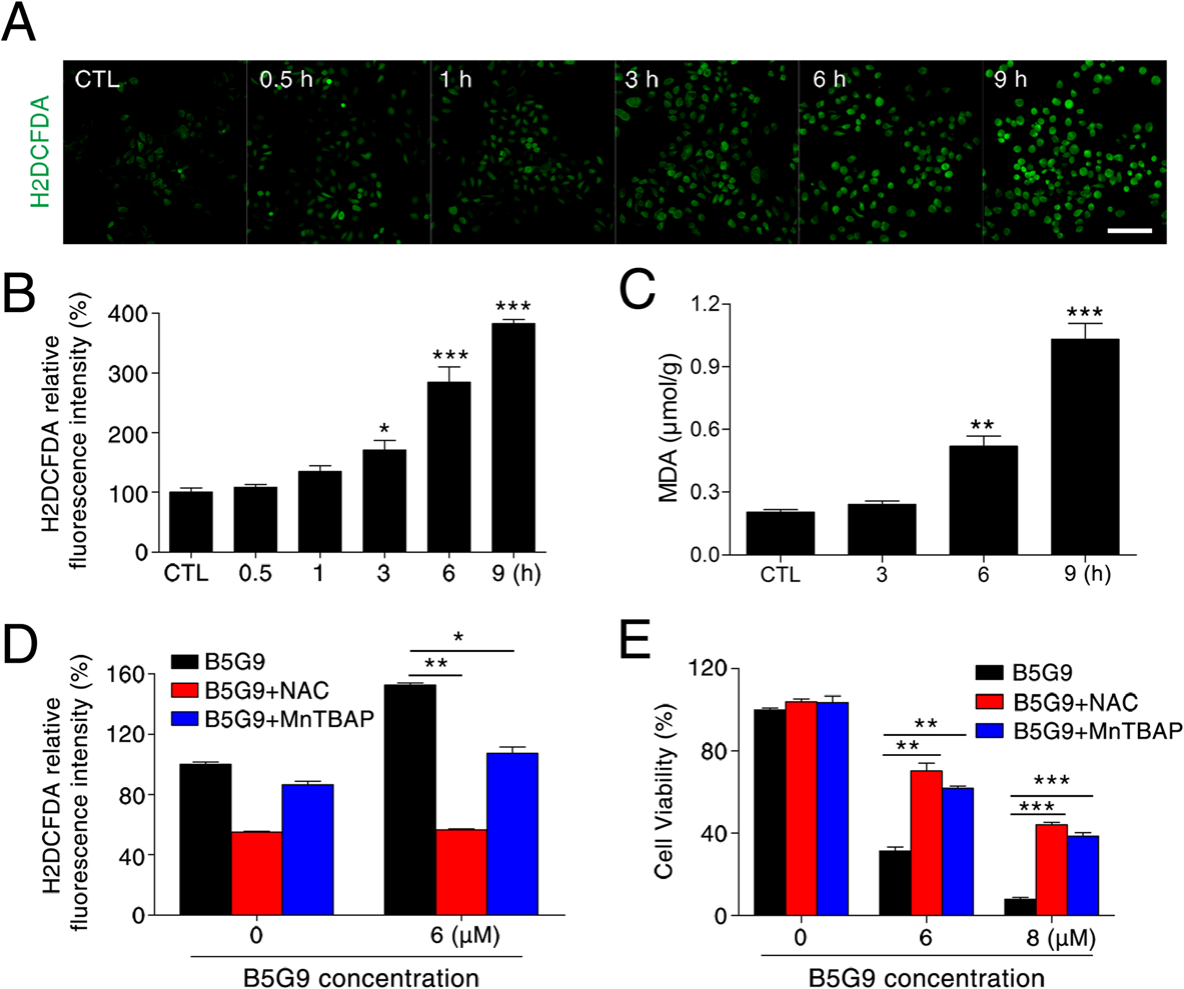
B5G9-induced apoptosis is ROS-dependent. **a** B5G9 elevated ROS level by a time-dependent manner in HepG2 cells. HepG2 cells were stained with H_2_DCFDA (10 μM) after being treated with B5G9 (6 μM) at the indicated times. The H_2_DCFDA fluorescence was observed by a fluorescence microscope. Original magnifications: 100 ×; scale bar: 200 μm. **b** The H_2_DCFDA fluorescence intensity of HepG2 cells treated with B5G9 at the indicated times was detected by a microplate reader. ^*^ *P* ≤ 0.05, ^***^ *P* ≤ 0.001 *vs* control. **c** B5G9 induced lipid peroxidation. After being treated with B5G9 (6 μM) for the indicated times, MDA level were detection by a Lipid Peroxidation MDA Assay Kit. ^**^ *P* ≤ 0.01, ^***^ *P* ≤ 0.001 *vs* control. **d** The elevated ROS level induced by B5G9 was abrogated by NAC or MnTBAP. HepG2 cells were treated with B5G9 (6 μM) in the presence or absence of NAC (20 mM) or MnTBAP (200 μM) for 3 h. The H_2_DCFDA fluorescence intensity was detected by a microplate reader. ^*^ *P* ≤ 0.05, ^**^ *P* ≤ 0.01. **e** NAC or MnTBAP alleviates B5G9-induced cell death. HepG2 cells were treated with B5G9 (6 μM) in the presence or absence of NAC (20 mM) or MnTBAP (200 μM) for 12 h. Cell viability was measured by MTT assay. ^**^ *P* ≤0.01, ^***^ *P* ≤ 0.001

ROS, which serve as a second messenger in cellular physiology, play an important role in apoptosis [41, 42]. To determine whether B5G9 could induce excess ROS production in HepG2 cells, we used a fluorescence probe, H_2_DCFDA. When the probe is oxidized by ROS, it transforms into its oxidized form, DCF, which emits bright green fluorescence. As shown in Fig. 3a and b, DCF fluorescence significantly increased after 3 h and further increased until 9 h after B5G9 treatment, indicating that B5G9 induces oxidative stress in HepG2 cells. As a consequence of oxidative stress, cells undergo protein oxidation, lipid peroxidation and DNA damage [43]. Malondialdehyde (MDA), a product of lipid peroxidation, was substantially increased after B5G9 treatment (Fig. 3c), which further confirms the occurrence of oxidative stress. To detect the role of ROS in B5G9-induced cell death, we pretreated HepG2 cells with N-acetyl cysteine (NAC) (an antioxidant) or MnTBAP (a SOD mimic) before B5G9 treatment. As shown in Fig. 3d and e, NAC or MnTBAP completely abrogated B5G9-induced ROS generation and significantly attenuated B5G9-induced cell death. These results showed that B5G9-induced apoptotic cell death is dependent on ROS elevation.

### Identification of ROS-associated genes using a Human Oxidative Stress Plus PCR Array

**Fig. 4:**
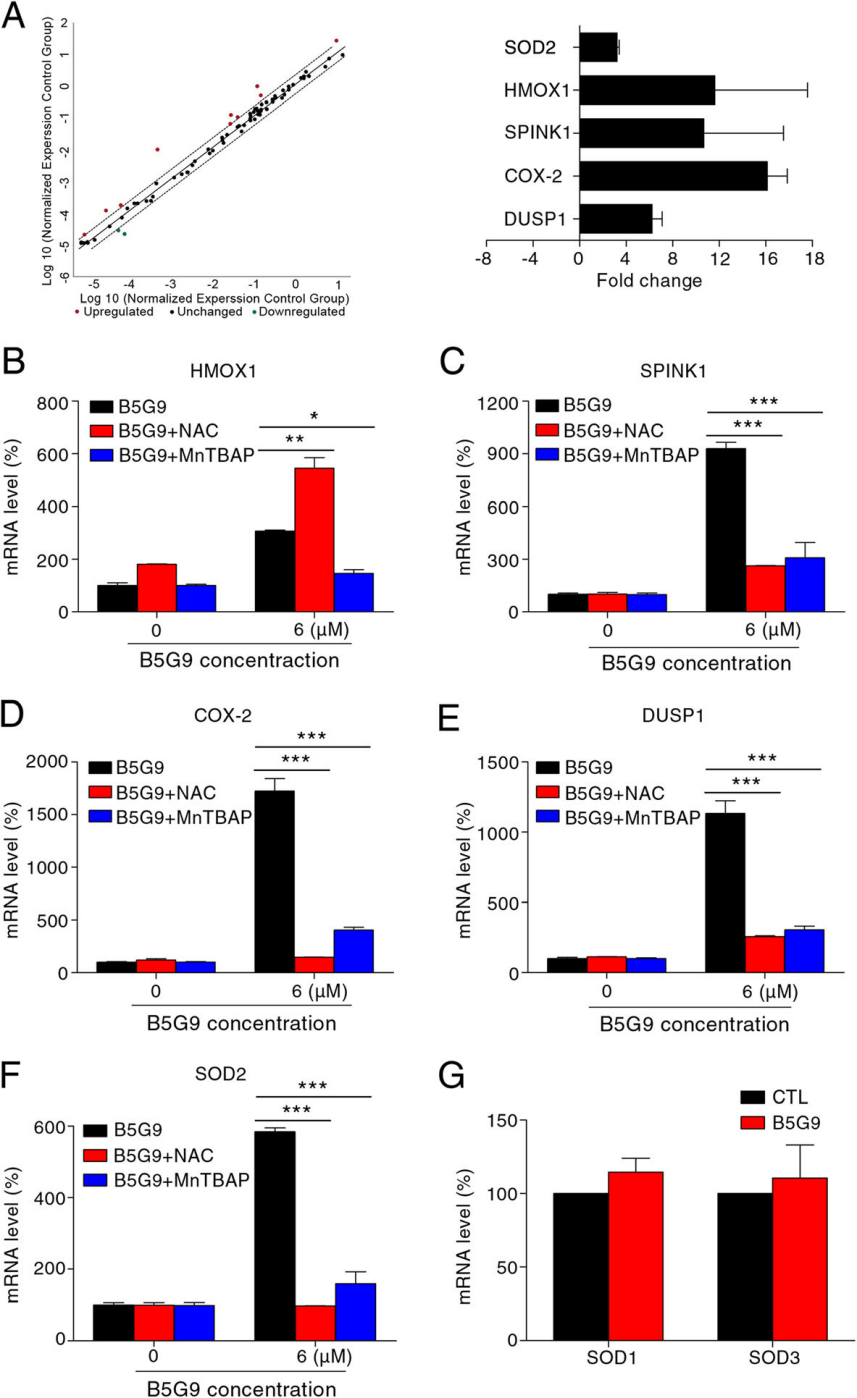
Identification of ROS-associated genes by Human Oxidative Stress Plus PCR Array. **a** The gene screening of oxidative stress-related proteins. HepG2 cells were treated with B5G9 (6 μM) for 3 h, the RT2 Profiler PCR Array was performed (left panel). The fold change values of these genes were calculated (right panel). The changes of HMOX1 (**b**), SPINK1 (**c**), COX-2 (**d**) DUSP1 (**e**) and SOD2 (**f**) gene levels were consequences of ROS overload. HepG2 cells were treated with B5G9 (6 μM) in the presence or absence of NAC (20 mM) or MnTBAP (200 μM) for 3 h, the fold changes of these genes were detected by RT-PCR. ^*^ *P* ≤ 0.05, ^**^ *P* ≤ 0.01, ^***^ *P* ≤ 0.001. **g** B5G9 had no effect on mRNA level of SOD1 and SOD3. HepG2 cells were treated with B5G9 (6 μM) in the presence or absence of NAC (20 mM) for 3 h, the fold changes of these genes were detected by RT-PCR

To further confirm oxidative stress induced by B5G9 and explore the mechanism of ROS burst, we used a Human Oxidative Stress Plus RT^2^ Profiler PCR Array to identify the changes in ROS-associated gene expression after B5G9 treatment. The results showed that five genes (the red dots) were upregulated after B5G9 treatment (Fig. 4a). However, the antioxidants NAC and MnTBAP completed the abrogated B5G9-induced upregulation of these genes (Fig. 4b-f), whereas NAC partly enhanced B5G9-induced HMOX1 upregulation (Fig. 4b). Thus, the upregulation of these genes is probably a stress response against oxidative stress. Indeed, these genes are sensitive to oxidative stress, and some of them can mediate cellular oxidative stress [44–47]. Interestingly, we found that only SOD2 was significantly upregulated by B5G9, whereas SOD1 and SOD3 showed no changes (Fig. 4g). SOD2 is a mitochondria-specific antioxidase, and its B5G9-mediated upregulation was completely inhibited by NAC and MnTBAP (Fig. 4g), implying that B5G9 may trigger mitochondrial oxidative stress.

### Mitochondria are the major source of B5G9-induced ROS production

**Fig. 5:**
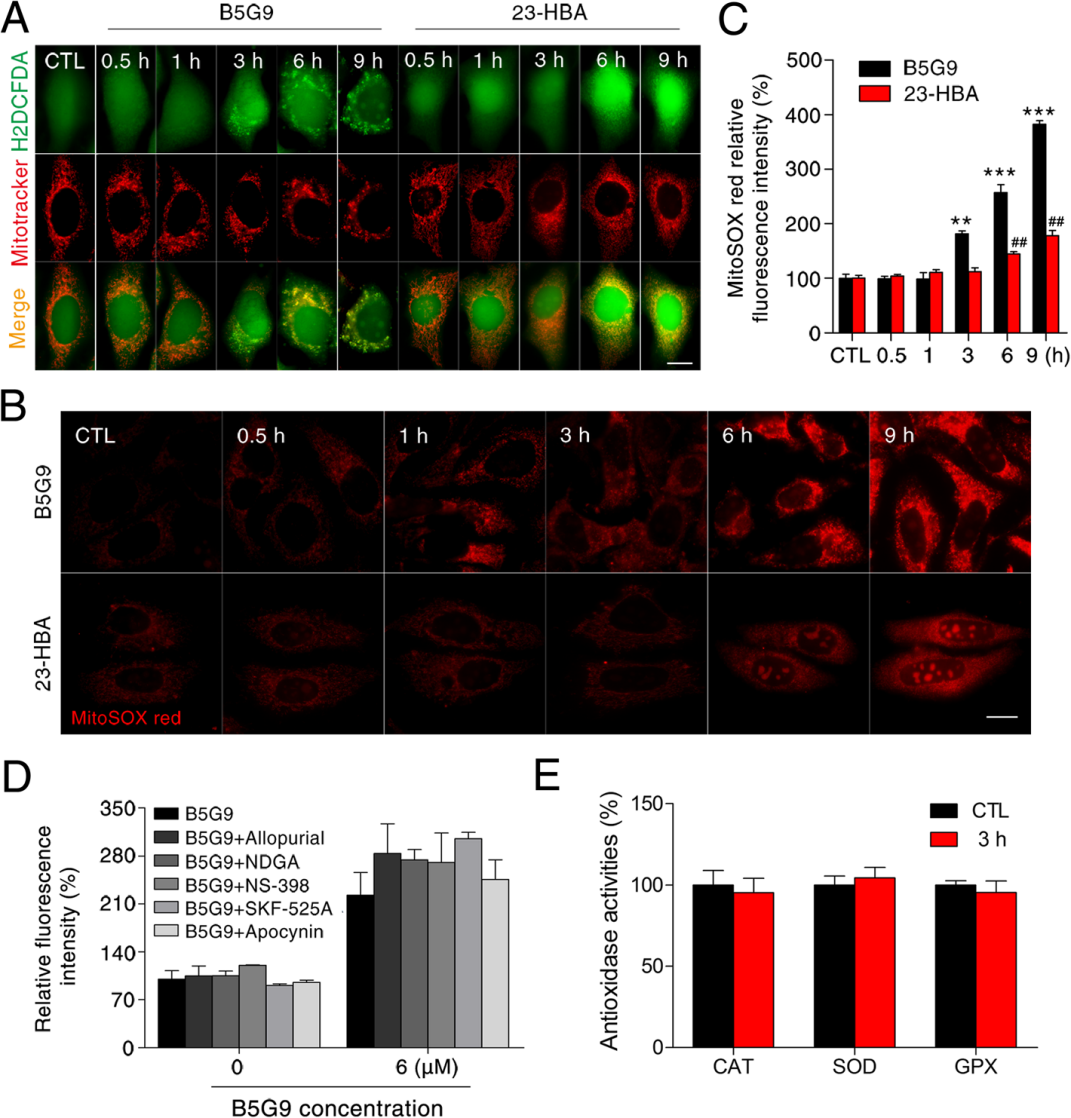
Mitochondria are the main source of B5G9-induced ROS overproduction. **a** B5G9-induced punctate H_2_DCFDA fluorescence co-localized with mitochondrial tracker. HepG2 cells were treated with B5G9 (6 μM) or 23-HBA (30 μM) for indicated times. Then cells were stained with H_2_DCFDA (10 μM) and mitotracker (100 nM) for 30 min. The fluorescence was observed by a fluorescence microscope. Original magnifications: 630 ×, scale bar: 10 μm. **b**-**c** B5G9 induced mitochondrial ROS overload. HepG2 cells were treated with B5G9 (6 μM) or 23-HBA (30 μM) for indicated times, and then cells were stained with mitoSOX (5 μM) red for 10 min. The fluorescence was observed by a fluorescence microscope (**b**). Original magnifications: 630 ×; scale bar: 10 μm. The fluorescence was also detected by a microplate reader (**c**), ^**^ *P* ≤ 0.01, ^***^ *P ≤* 0.001, B5G9 *vs* control, ^##^ *P* ≤ 0.01, 23-HBA *vs* control. **d** B5G9-induced elevated ROS was independent of xanthine oxidase, lipoxygenase, cyclooxygenase, cytochrome p450 or NADPH oxidase. HepG2 cells were pretreated with allopurinol (10 μM), NDGA (10 μM), NS-398 (10 μM), SKF-525A (10 μM) or apocynin (20 μM), followed by B5G9 (6 μM) treatment for 3 h, the H_2_DCFDA fluorescence intensity was detected by a microplate reader. **e** B5G9 had no influence on activities of CAT, SOD and GPx. After being treated with B5G9 (6 μM) treatment for 3 h, the activities of antioxidases were measured by detection kits

Mitochondria are the main intracellular location for oxygen consumption and energy generation, and they are also an important source of ROS. We found that the punctate fluorescence of H_2_DCFDA induced by B5G9 (3, 6 and 9 h) can co-localize with a mitochondria-selective probe, indicating that elevated ROS may be produced by the mitochondria, and similar characteristics were also found in 23-HBA-treated HepG2 cells (Fig. 5a). MitoSOX Red, a mitochondria-targeted ROS probe, was used to further confirm these results. As expected, B5G9 significantly increased MitoSOX Red fluorescence from 3 to 9 h, whereas 23-HBA had a weak effect (Fig. 5b and c). In addition to the mitochondria, xanthine oxidase, lipoxygenase, cyclooxygenase, cytochrome p450 and NADPH oxidase are sources of cellular ROS [48–51]. However, inhibitors of these enzymes failed to block B5G9-induced ROS generation (Fig. 5d). Impairment of antioxidant enzymes, such as catalase (CAT), SOD, and glutathione peroxidase (GPx), is known to induce ROS production. However, the activities of CAT, SOD and GPx were not inhibited (Fig. 5e). Taken together, our results indicated that B5G9 induced mitochondrial ROS generation and had no effect on other ROS-producing enzymes and antioxidant enzymes.

### B5G9 induces mitochondrial dysfunction

**Fig. 6:**
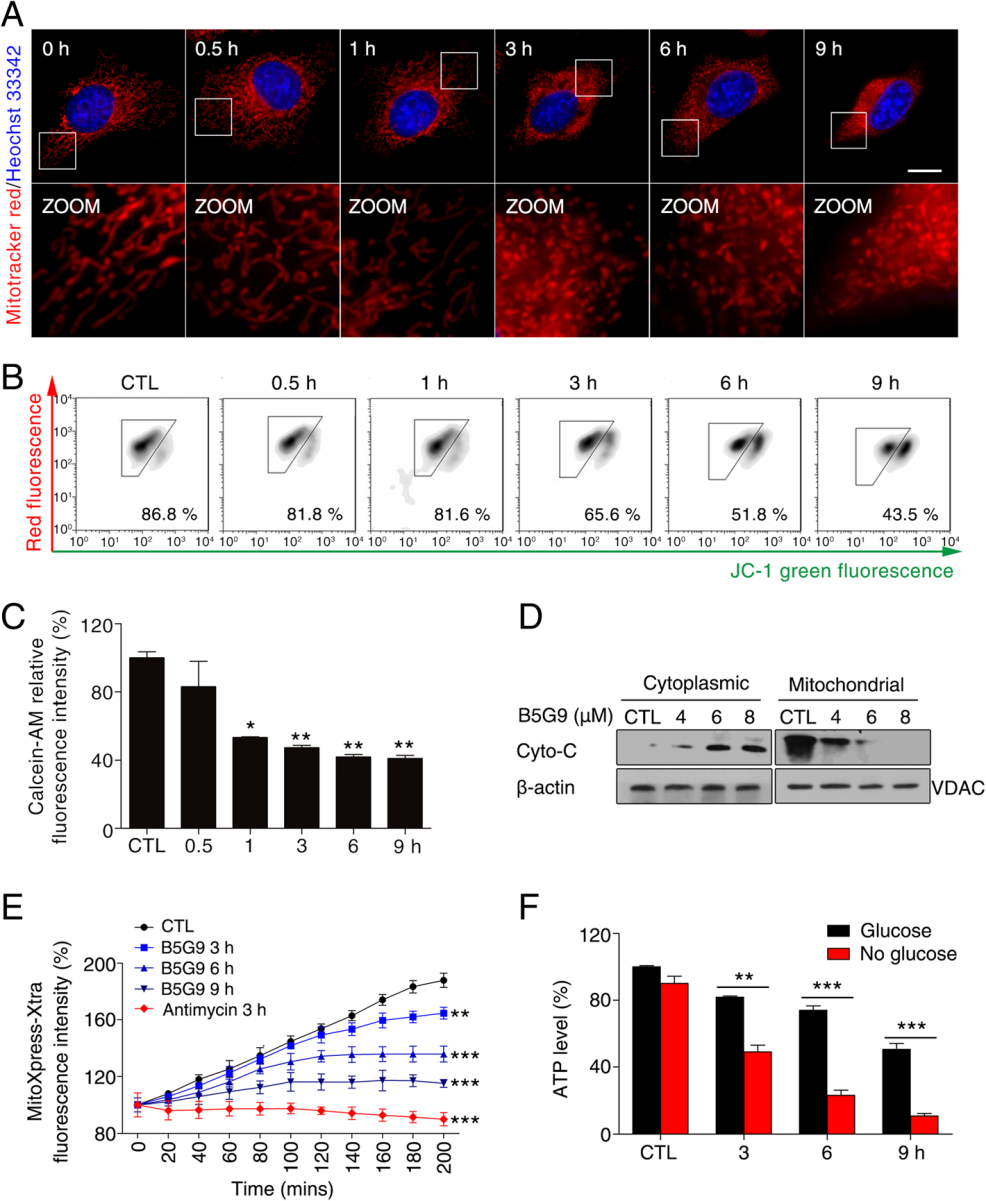
B5G9 treatment induces mitochondrial dysfunction. **a** B5G9 induced mitochondrial morphology changes. HepG2 cells treated with B5G9 (6 μM) for indicated times were stained with MitoTracker Red CMXRos (100 nM) for 20 min, the cells were observed by a fluorescence microscope. Original magnifications: 630 ×; scale bar: 10 μm, (**b**) B5G9 induced mitochondrial membrane potential collapse. HepG2 cells were treated with B5G9 (6 μM) for indicated times, and then stained with JC-1 (5 μM) fluorescence dye, and changes of *ΔΨm* were determined by flow cytometry. **c** B5G9 increased mitochondrial membrane permeabilization. HepG2 cells were treated with B5G9 (6 μM) for indicated times, then cells were incubated with calcein-AM for 30 min, the fluorescence was detected by a microplate reader, ^*^ *P* ≤ 0.05, ^**^ *P* ≤ 0.01 *vs* control. **d** Cyto *c* released from mitochondria to cytoplasm after B5G9 treatment. After being treated with various concentrations of B5G9 for 24 h, HepG2 cells lysate was divided into mitochondrial fraction and cytoplasmic fraction and detected by western bolt, β-actin and VDAC were used as loading controls for cytoplasm and mitochondria respectively. **e** O_2_ consumption rate was inhibited by B5G9. HepG2 cells were treated with B5G9 (6 μM) for 3, 6, 9 h and antimycin (6 μM) for 3 h, the O_2_ consumption rate was measured by a MitoXpress-Xtra-based assay. ^**^ *P* ≤ 0.01, ^***^ *P ≤* 0.001 *vs* control. **f** B5G9 impaired mitochondrial ATP production. HepG2 cells were treated with B5G9 (6 μM) for 3, 6 and 9 h in complete medium or no-glucose medium, the ATP level was detected by a CellTiter-Glo Luminescent Assay, ^**^ *P* ≤ 0.01, ^***^ *P* ≤ 0.001

Excessive ROS production in the mitochondria leads to mitochondrial dysfunction. Next, we investigated the mitochondrial function following B5G9 treatment. Morphological observation was performed with a Mito-Tracker staining assay. As shown in Fig. 6a, in untreated HepG2 cells, filiform mitochondria were observed. By contrast, mitochondria in HepG2 cells treated with B5G9 shrank, swelled and fragmented, indicating mitochondrial dysfunction. *ΔΨm* collapse, a hallmark of mitochondrial dysfunction, was induced by B5G9 in a time-dependent manner (Fig. 6b). In addition, B5G9 decreased the calcein-AM fluorescence, indicating the permeabilization of the mitochondrial membrane (Fig. 6c). This result was further confirmed by cytochrome *c* translocation from the mitochondria to the cytoplasm (Fig. 6d), which is considered a major event of the mitochondrial apoptotic pathway. As mitochondria are the main location for aerobic respiration for energy production, we next assessed the effect of B5G9 on OCR and ATP production. As shown in Fig. 6e, B5G9 decreased OCR in a time-dependent manner from 3 to 9 h, and antimycin, an inhibitor of complex III, was used as a positive control. Moreover, the cellular ATP levels of HepG2 cells maintained in no-glucose medium were significantly decreased upon B5G9 treatment (Fig. 6f), indicating that B5G9 suppressed mitochondrial ATP production, because HepG2 cells can only produce ATP by mitochondria when glycolysis is inhibited by withdrawing glucose. These results suggest that B5G9 induces mitochondrial dysfunction.

### Mitochondrial DNA-depleted HepG2 cells are resistant to B5G9 treatment

**Fig. 7:**
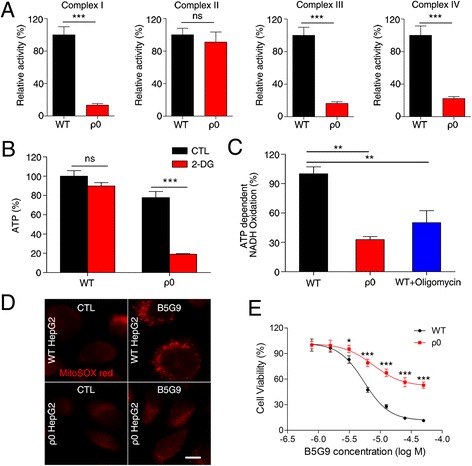
ρ0 HepG2 cells were less sensitive to B5G9 treatment. **a** Mitochondrial complex activities of wt HepG2 or ρ0 HepG2 were detected by related assay kits, ^***^ *P* ≤ 0.001. **b** ATP production of ρ0 HepG2 cells was significantly decreased in the present of 2-DG. After being treated with 2-DG (2 mM) for 2 h, ATP level of wt HepG2 or ρ0 HepG2 was measured by a CellTiter-Glo Luminescent Assay, ^***^ *P* ≤ 0.001. **c** ρ0 HepG2 had a lower ATP synthetase activity than wt HepG2. The ATP synthetase activity of wt HepG2 treated in the present or absent of oligomycin (6 μM) and ρ0 HepG2 was measured by a cayman ATP synthetase activity kit, ^**^ *P* ≤ 0.01. **d** ρ0 HepG2 cells had no significant change in mitoSOX red fluorescence after B5G9 treatment, original magnifications : 630 ×; scale bar: 10 μm. **e** ρ0 HepG2 cells were less sensitive to B5G9 treatment. Being treated with various concentrations of B5G9 for 12 h, cell viability was measured by MTT assay. ^*^ *P* ≤ 0.01, ^***^ *P* ≤ 0.001

To determine whether mitochondria are essential for B5G9-induced ROS-dependent cell death, we generated mitochondrial DNA-depleted ρ0 HepG2 cells via treatment with EB, pyruvate and uridine. ATP detection and mitochondrial complex activity assays were performed to characterize the depletion of mtDNA in ρ0 HepG2 cells. The activities of mitochondrial complex I, III and IV, but not complex II, were significantly decreased in ρ0 HepG2 cells (Fig. 7a). This difference occurred because all proteins of complex II are encoded by genomic DNA, whereas other complex proteins are encoded by genomic DNA as well as mitochondrial DNA [52]. As a result of mitochondrial complex subunit depletion, ATP levels in HepG2 cells treated with 2-deoxyglucose (2-DG), an inhibitor of glycolysis, significantly decreased (Fig. 7b) due to the impairment of ATPase activity (Fig. 7c), but in the absence of 2-DG, ATP levels showed no significant difference between wt and ρ0 HepG2 cells because ρ0 HepG2 cells can use glycolysis to survive in the presence of pyruvate and uridine. Moreover, MitoSOX Red fluorescence was markedly elevated in wt HepG2 cells treated with B5G9, whereas the fluorescence in ρ0 HepG2 cells only slightly increased (Fig. 7d), which further indicated that B5G9-induced ROS generation was derived from mitochondria. The sensitivity of wt and ρ0 HepG2 cells to B5G9 treatment was measured with MTT assays. As expected, ρ0 HepG2 cells were resistant to B5G9 (Fig. 7e), indicating that mitochondria play an important role in B5G9-induced ROS-dependent cell death.

## Discussion

Traditional Chinese medicine (TCM) is an abundant source of anti-HCC agents. Recently, the modification of natural products from TCM has become a promising approach for novel drug development. To enhance the antitumour efficacy of 23-HBA and reduce its side effects, we performed a series of structural modifications of 23-HBA and conducted a bioactivity screening [24]. We found that a piperazidine derivative of 23-HBA, B5G9, showed excellent anti-HCC activity both in vitro and in vivo.

In the current study, we evaluated the anti-HCC activity of B5G9 on four HCC cell lines, including HepG2, Bel-7402 and Hep3B cells (p53-null), and multidrug-resistant HepG2/ADM cells. B5G9 showed comparable cytotoxicity in the four hepatoma cell lines, which indicates the potential of B5G9 in the treatment of various types of HCC cells, regardless of p53 status. B5G9 also exhibited outstanding anti-proliferation activity on HepG2 tumour xenografts without significant toxicity, whereas 23-HBA had a minimal effect.

ROS generation is considered the key mechanism for the anti-cancer activity of BA and 23-HBA [27, 28, 53]. However, the source of ROS was unclear. In the Human Oxidative Stress Plus PCR Array, we surprisingly found that B5G9-induced mitochondria-specific SOD2 upregulation was completely inhibited by antioxidants. The results indicated that the mitochondria might undergo oxidative stress. This hypothesis was confirmed by the co-localization of H_2_DCFDA and Mito-Tracker as well as MitoSOX Red staining. We found that 23-HBA had a similar but weaker effect, which could explain why B5G9 had greater anti-HCC activity than that of 23-HBA; however, B5G9 may have a different underlying mechanism of mitochondrial dysfunction. Moreover, we noted that B5G9 just induced a slight increase of mitochondrial ROS and apoptosis in normal liver LO2 cells compare to HepG2 cells (Additional file 2: Figure S1A&B) which indicated its selective cytotoxicity against HCC cells. These results also implied the important role of mitochondrial ROS in B5G9-induced apoptosis. In addition to mitochondria, NADPH oxidase, XO, LOX, COX and cytochrome p450 contribute to ROS overproduction, but the results showed that inhibitors of these enzymes failed to abolish B5G9-induced ROS production. All these data indicate that mitochondria are the major site of B5G9-induced ROS production. Inhibition of mitochondrial complex activity (especially complex I and III) can induce ROS production [54, 55]. We further measured mitochondrial complex activities upon B5G9 treatment by cell-based assay. As shown in Additional file 2: Figure S1C, B5G9 treatment (0.5, 1 and 3 h) had no effect on activities of mitochondrial complex I, III and V. In our study, B5G9-induced mitochondrial-ROS burst was observed at 3 h. These data indicate that B5G9-induced mitochondrial-ROS overload was probably not a result of mitochondrial complex complex inhibition. The mechanisms underlying B5G9-induced mitochondrial-ROS burst are complicated and need be explored in our future study. Recently, BA was found to induce ROS production in hepatocellular carcinoma via a p53-dependent p66^shc^/miR-21-Sod2 pathway [53]. However, B5G9 could also induce oxidative stress in Hep3B cells (Additional file 2: Figure S1D), indicating that a p53-independent mechanism underlying the ROS burst induced by B5G9 must exist. Wolfgang Wick found that BA-induced ROS generation required new protein synthesis [27], but this was not observed in our studies because cycloheximide (CHX), an inhibitor of *de novo* protein synthesis, could not inhibit the ROS induced by B5G9 (Additional file 2: Figure S1E&F). This result was also confirmed by the fact that five genes (HMOX1, SPINK1, COX-2, DUSP1 and SOD2) were significantly upregulated by B5G9, and their upregulation was a consequence of ROS production (Fig. 4). Dual-specificity phosphatase 1 (DUSP1) is a negative regulator of MAPKs that dephosphorylates both the threonine/serine and tyrosine residues of the substrates to facilitate tumourigenesis [56]. In human HCC, the activated RAS-MAPK cascade leads to ERK activation, which results in the phosphorylation of its inhibitor, DUSP1. Then, the phosphorylated DUSP1 is degraded by the ubiquitin proteasome system [57]. Hence, DUSP1 is negatively correlated with ERK activity and acts as a negative regulator of HCC development [58]. Thus, DUSP1 upregulation may be a promising approach in HCC therapy. In our study, we found that the DUSP1 mRNA level was substantially upregulated; however, the role of DUSP1 in B5G9-induced HepG2 cell death requires further study.

It has been frequently reported that BA as well as its derivatives activated the mitochondrial apoptotic pathway. Unfortunately, few studies have examined the requirement of mitochondria for their anti-cancer effects. To gain insight into this issue, we established a mitochondrial DNA-depleted ρ0 HepG2 cell line. B5G9 only slightly increased the fluorescence of MitoSOX Red in ρ0 HepG2 cells, and ρ0 HepG2 cells were less sensitive to the B5G9 treatment compared with wt HepG2 cells. These phenomena are partly due to the incomplete efficiency of mitochondrial DNA depletion. Nevertheless, these results indicate that mitochondria play a crucial role in B5G9-induced cell death. MPT is a key event in the mitochondrial apoptotic pathway. BA-induced MPT was a consequence of the opening of the mitochondrial PT pore, which was abolished by cyclosporine A (CsA) or bongkrekic acid [59, 60]. In contrast to these data, we found that cyclosporine failed to alleviate B5G9-induced MPT and cell death (Additional file 2: Figure S1G&H). Recently, a novel mechanism associated with mitochondrial cardiolipin was discovered. BA induced cancer cell death by modifying the biosynthesis of cardiolipin . This modification was accomplished by the inhibition of the activity of steroyl-CoA-desaturase [61]. Moreover, BA could also induce MPT by directly interacting with the mitochondrial membrane to change the permeability of the cardiolipin film [62]. These results suggested that piperazidine may enhance the mitochondrial cardiolipin modification effect, and this should be further investigated.

## Conclusion

In summary, we discovered a piperazidine derivative of 23-HBA, B5G9, with excellent anti-HCC effects both in vivo and in vitro, and no obvious toxic effects. The underlying mechanism was associated with mitochondria-derived ROS production, and we further identified the importance of the mitochondria in B5G9-induced cell death. This study identifies a potential agent for anti-HCC therapy and elucidates the source of ROS induced by BA and its derivatives.

## Additional files

Additional file 1: Table S1. Primer sequences of oxidative stress-related genes. (DOCX 16 kb)
Additional file 2: Figure S1. (A) Effect of B5G9 on mitochondrial-ROS production of LO2 cells. HepG2 and LO2 cells were treated with B5G9 (6 μM) for indicated times, and then cells were stained with mitoSOX (5 μM) red for 10 min. The fluorescence was detected by a microplate reader, ^***^ *P ≤* 0.001, B5G9 *vs* control (HepG2). (B) Effect of B5G9 on cell apoptosis of LO2 or HepG2 cells. HepG2 or LO2 cells were treated with B5G9 (6 and 8 μM) for 24 h. Apoptotic rate (PI positive cells plus Annexin V positive cells) was measured by PI/Annexin V assay. ^**^ *P* ≤0.01. (C) B5G9 had no effect on mitochondrial complex I, III and V activities. Mitochondrial protein of HepG2 cells treated with B5G9 (6 μM) for 0.5, 1 and 3 h were extracted, and then activities of mitochondrial complex I, III and V were detected using related detection kit. ^*^ *P* ≤ 0.05, ^**^ *P* ≤ 0.01 *vs* control. (D) B5G9 induced ROS overload in Hep3B cells in a time-dependent manner. Hep3B cells were stained with H_2_DCFDA (10 μM) after being treated with B5G9 (6 μM) for indicated times. The fluorescence of H_2_DCFDA was measured by a microplate reader. ^***^ *P* ≤ 0.001 *vs* control. (E) B5G9 induced ROS overload was protein *de novo* synthesis independent. HepG2 cells were exposed with B5G9 (6 μM) for 6 h after pretreatment with CHX (10 μM). The fluorescence of H_2_DCFDA was observed by a fluorescence microscope and measured by a microplate reader (F). Original magnifications: 200 ×; scale bar: 50 μm. (G) B5G9 induced mitochondrial membrane permeabilization was PT pore-independent. HepG2 cells were treated with B5G9 (6 μM) in the presence or absence of CsA (5 μM) for 6 h, then cells were incubated with calcein-AM for 30 min, the fluorescence was detected by a microplate reader. (H) CsA could not rescue B5G9 induced cell death in HepG2 cells. HepG2 cells were treated with B5G9 in the presence or absence of CsA (5 μM) for 12 h, cell viability was detected by MTT assay. (TIF 2973 kb)

## Abbreviations

23-HBA: 23-hydroxy betulinic acid
2-DG: 2-deoxyglucose
ALT: Alanine transaminase
AST: Aspartate aminotransferase
BA: Betulinic acid
BUN: Blood urea nitrogen
CAT: Catalase
CHX: Cycloheximide
CK: Creatine kinase
CsA: Cyclosporine A
DUSP1: Dual-Specificity Phosphatase 1
ETC: Electron transport chain
GPx: Glutathione peroxidase
HCC: Hepatocellular carcinoma
HGB: Hemoglobin
MDA: Methane dicarboxylic aldehyde
MPT: Mitochondrial permeability transition
NAC: N-acetyl cysteine
OCR: O_2_ consumption rate
PLT: Platelet
RBC: Red blood cell
ROS: Reactive oxygen species
SOD: Superoxide dismutase
TBA: Thiobarbituric acid
TCM: Traditional Chinese medicine
WBC: White blood cell
