# Supplementary material for: A piperazidine derivative of 23-hydroxy betulinic acid induces a mitochondria-derived ROS burst to trigger apoptotic cell death in hepatocellular carcinoma cells
Source: J Exp Clin Cancer Res. 2016 Dec 8;35:192. doi: 10.1186/s13046-016-0457-1 (PMC5146873; doi:10.1186/s13046-016-0457-1)
Supplement: Additional file 1: Table S1. — Primer sequences of oxidative stress-related genes. (DOCX 16 kb) [file 13046_2016_457_MOESM1_ESM.docx]

Primer sequences of oxidative stress-related genes

| Gene | | Primer sequence |
| --- | --- | --- |
| HMOX1 | Forward | TCCTGGCTCAGCCTCAAATG |
|  | Reverse | CGTTAAACACCTCCCTCCCC |
| SPINK1 | Forward | TATAGCCCAGTAGGTGGGGC |
|  | Reverse | AAGTTCTGCGTCCAGAGGTC |
| COX-2 | Forward | CGGTGAAACTCTGGCTAGACAG |
|  | Reverse | GCAAACCGTAGATGCTCAGGGA |
| DUSP1 | Forward | CCCGAGTTCCTCTGGGTTTC |
|  | Reverse | CCGGATCACACACTGAGTCC |
| SOD1 | Forward | CTCACTCTCAGGAGACCATTGC |
|  | Reverse | CCACAAGCCAAACGACTTCCAG |
| SOD2 | Forward | GGGAGCACGCTTACTACCTT |
|  | Reverse | GCTTACTGTATTCTGCAGTACTCT |
| SOD3 | Forward | AGGTCTCACCTTCGCCTTTG |
|  | Reverse | TCAGACCTACTGAGTGGGGG |
| GAPDH | Forward | AGGTCGGTGTGAACGGATTTG |
|  | Reverse | TGTAGACCATGTAGTTGAGGTCA |
