# Supplementary figures and images for: A piperazidine derivative of 23-hydroxy betulinic acid induces a mitochondria-derived ROS burst to trigger apoptotic cell death in hepatocellular carcinoma cells
Source: J Exp Clin Cancer Res. 2016 Dec 8;35:192. doi: 10.1186/s13046-016-0457-1 (PMC5146873; doi:10.1186/s13046-016-0457-1)

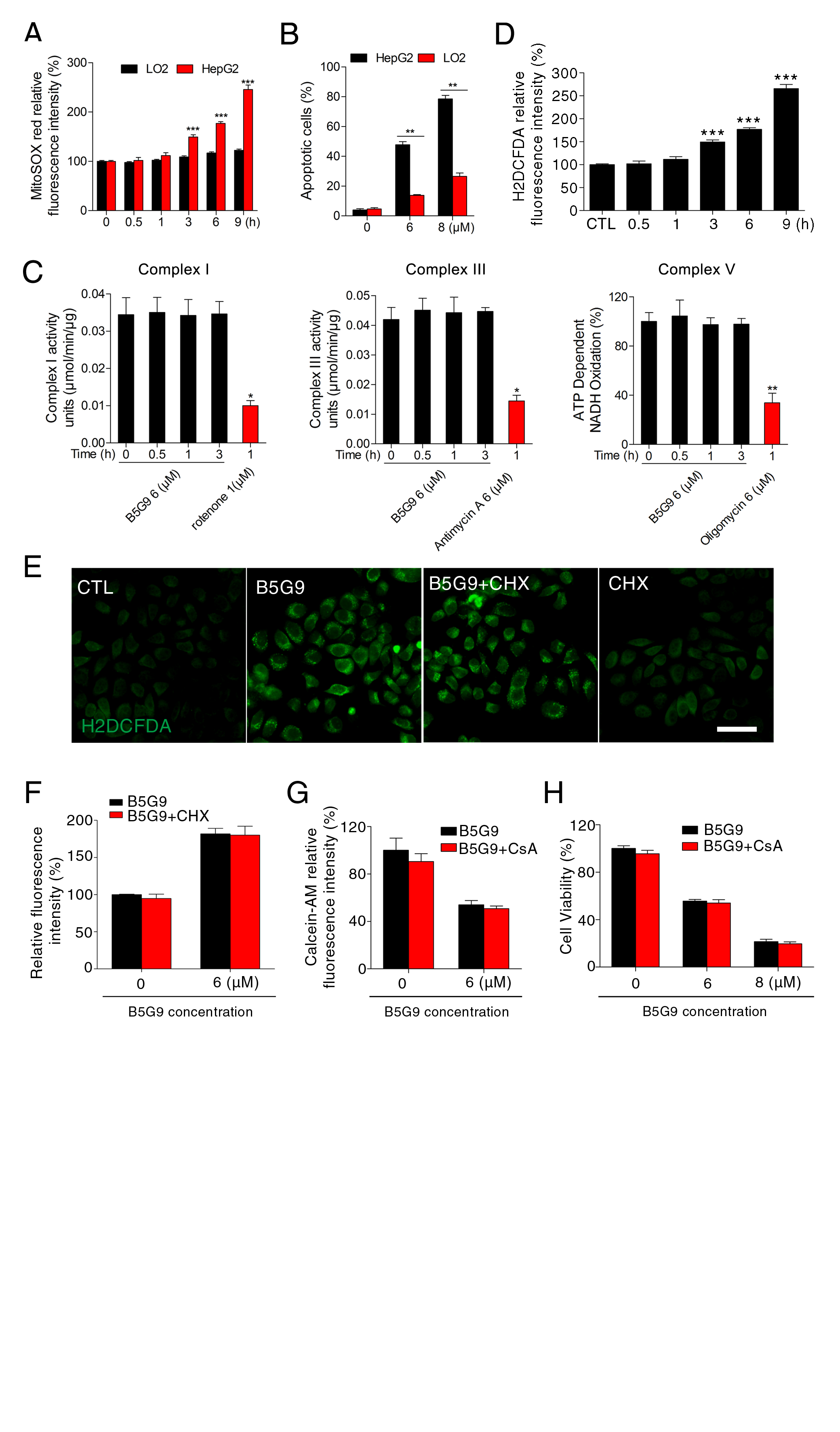

Supplement: Additional file 2: Figure S1. — (A) Effect of B5G9 on mitochondrial-ROS production of LO2 cells. HepG2 and LO2 cells were treated with B5G9 (6 μM) for indicated times, and then cells were stained with mitoSOX (5 μM) red for 10 min. The fluorescence was detected by a microplate reader, *** P ≤ 0.001, B5G9 vs control (HepG2). (B) Effect of B5G9 on cell apoptosis of LO2 or HepG2 cells. HepG2 or LO2 cells were treated with B5G9 (6 and 8 μM) for 24 h. Apoptotic rate (PI positive cells plus Annexin V positive cells) was measured by PI/Annexin V assay. ** P ≤0.01. (C) B5G9 had no effect on mitochondrial complex I, III and V activities. Mitochondrial protein of HepG2 cells treated with B5G9 (6 μM) for 0.5, 1 and 3 h were extracted, and then activities of mitochondrial complex I, III and V were detected using related detection kit. * P ≤ 0.05, ** P ≤ 0.01 vs control. (D) B5G9 induced ROS overload in Hep3B cells in a time-dependent manner. Hep3B cells were stained with H2DCFDA (10 μM) after being treated with B5G9 (6 μM) for indicated times. The fluorescence of H2DCFDA was measured by a microplate reader. *** P ≤ 0.001 vs control. (E) B5G9 induced ROS overload was protein de novo synthesis independent. HepG2 cells were exposed with B5G9 (6 μM) for 6 h after pretreatment with CHX (10 μM). The fluorescence of H2DCFDA was observed by a fluorescence microscope and measured by a microplate reader (F). Original magnifications: 200 ×; scale bar: 50 μm. (G) B5G9 induced mitochondrial membrane permeabilization was PT pore-independent. HepG2 cells were treated with B5G9 (6 μM) in the presence or absence of CsA (5 μM) for 6 h, then cells were incubated with calcein-AM for 30 min, the fluorescence was detected by a microplate reader. (H) CsA could not rescue B5G9 induced cell death in HepG2 cells. HepG2 cells were treated with B5G9 in the presence or absence of CsA (5 μM) for 12 h, cell viability was detected by MTT assay. (TIF 2973 kb) [file 13046_2016_457_MOESM2_ESM.tif]
